# Understanding the social–emotional components of our “number sense”: insights from a novel non-symbolic numerical comparison task

**DOI:** 10.3389/fpsyg.2024.1175591

**Published:** 2024-03-05

**Authors:** Marta K. Mielicki, Rahma Mbarki, Jinjing Jenny Wang

**Affiliations:** ^1^Center for Cognitive Science, Rutgers University–New Brunswick, New Brunswick, NJ, United States; ^2^Department of Psychology, Rutgers University–New Brunswick, New Brunswick, NJ, United States

**Keywords:** math anxiety, approximate number system, information seeking, confidence judgments, gender

## Abstract

**Introduction:**

A large body of work has identified a core sense of number supported by the Approximate Number System (ANS) that is present in infancy and across species. Although it is commonly assumed that the ANS directly processes perceptual input and is relatively independent from affective factors, some evidence points at a correlation between ANS performance and math anxiety. However, the evidence is mixed. We tested whether giving participants active control in completing a numerical task would change the relationship between math anxiety on performance.

**Methods:**

Adult participants (N = 103) completed a novel four-alternative-forced-choice non-symbolic numerical comparison task. In a repeated-measures design, participants either passively viewed different dot arrays or actively chose to view each array (i.e., active information-seeking) before deciding on the largest quantity. Participants also provided confidence judgments during the passive version of the task.

**Results:**

We replicated the ratio-dependent signature in participants’ accuracy in both the passive and active versions of the task using this novel paradigm, as well as in trial-level confidence judgments and information-seeking behavior. Participants’ self-reported math anxiety significantly correlated with their accuracy on the passive version of the task. Critically, the correlation disappeared in the active version of the task. Gender also emerged as a predictor of confidence judgments and a moderator of the effect of task on overall accuracy and the effect of active information seeking on accuracy in the active version of the task. Exploratory analysis of estimated Weber Fraction suggests that these results may be driven by auxiliary factors instead of changes in ANS acuity.

**Conclusion:**

These findings have implications for understanding the relationship between math anxiety and performance on numerical tasks.

## Introduction

We often encounter math and number problems under pressure — be it the time and mental pressure from a pop quiz or a final exam, or the social pressure when calculating a tip, or even choosing the right line to follow at a grocery store. Such pressure may relate to anxiety when thinking about numbers, which can impact how we learn and reason about numerical information. Can we alleviate the pressure by offering more control over a numerical decision-making process? The current study uses our intuitive “number sense” as a case study to investigate how active control may moderate the link between people’s math anxiety and numerical performance.

### The approximate number system and its covarying factors

A large body of work has identified a core sense of number supported by the Approximate Number System (ANS) that is present in infancy (Izard et al., 2009) and across species (Cantlon et al., 2009). The ANS supports the “number sense” to automatically represent large quantities (e.g., the number of dots in an array) without counting (Dehaene, 2011). Although the ANS is thought to underlie the ability to represent non-symbolic quantities, the basic number sense supported by ANS has been shown to relate to symbolic math performance controlling for age, SES, and other cognitive abilities (Libertus et al., 2011; Halberda et al., 2012; van Marle et al., 2018), and experimental evidence also supports a causal link between ANS and symbolic math skills (Wang et al., 2016, 2021). Like other perceptual systems, the ANS follows Weber’s Law – the ratio between numerical quantities determines how easy it is to distinguish them (Barth et al., 2003; Piazza et al., 2004). ANS task performance can be impacted by perceptual factors (Clayton et al., 2015) such as contour length (Clearfield and Mix, 2001), surface area (Feigenson et al., 2002), convex hull (Gebuis and Gevers, 2011), stimulus diameter (Sophian, 2007) or a combination of these factors (Gebuis and Reynvoet, 2012). In children, other manipulations, such presenting numerical comparison trials in order of increasing or decreasing difficulty, can also impact task performance (Odic et al., 2014; Wang et al., 2016, 2018, 2021).

In addition to these perceptual factors and task features, inducing negative emotional states through experimental manipulations can also affect performance on tasks thought to tap the ANS. For instance, presenting emotional stimuli (e.g., angry faces) during an estimation task resulted in underestimations of approximate quantities relative to a baseline condition with no emotional stimuli (Young and Cordes, 2013). Similarly, completing a numerical discrimination task under a threat condition in which the to-be-estimated quantities were images of spiders led to worse performance than a neutral condition (Hamamouche et al., 2017). A related line of work suggests that numerical discrimination involving the ANS is also subject to the influence of perceived social threat. Gonzalez et al. (2021) found that a stereotype threat manipulation (presenting the ANS task as a math task as opposed to a neutral task) led to worse performance for girls but did not impact boys.

### Math anxiety and the approximate number system

Taken together, this work suggests that although the ANS is a fundamental cognitive capacity, it may be subject to the influence of emotional and social factors. One such factor is math anxiety (MA), a feeling of tension or apprehension specifically related to engaging with mathematical tasks (Ashcraft, 2002). A large body of research has shown that MA is negatively related to math performance and achievement (Richardson and Suinn, 1972, Dowker, 2019, Mammarella et al., 2019; see meta-analyses by Hembree, 1990; Ma and Kishor, 1997; Namkung et al., 2019; Barroso et al., 2021). Since people with higher math anxiety typically have lower math achievement and performance, math anxiety has important implications in education, but also for general numeracy as it relates to everyday life (Choi et al., 2020) and for reasoning with health-related numerical information (Thompson et al., 2021).

Math anxiety may also play a role in the gender disparities that plague STEM fields since, in general, females tend to report higher MA than males (Else-Quest et al., 2010; Devine et al., 2012; Hart and Ganley, 2019). Similarly, males report more positive math attitudes (Sidney et al., 2021; Mielicki et al., 2022), higher math-related self-concept and expectations for success in math (Wigfield et al., 1997; Else-Quest et al., 2013), and are more likely to aspire to STEM careers than females (Lauermann et al., 2017).

The bulk of the existing research on MA has focused on symbolic mathematics, such as the kind that is typically taught in school (Dowker, 2019). In fact, MA may be most likely to impact performance on complex or unfamiliar math tasks (Maloney and Beilock, 2012). Since the ANS is thought to directly process perceptual input, it might be expected that ANS task performance should be relatively independent from affective factors such as MA. However, some evidence points to a relationship between MA and performance on tasks that are thought to tap basic magnitude representation, including ANS tasks. Some findings suggest that individuals with higher MA may represent symbolic numerical magnitude less precisely than those with lower MA (Maloney et al., 2011; Núñez-Peña and Suárez-Pellicioni, 2014). Other work specifically with non-symbolic ANS tasks has found a negative relationship between MA and ANS performance (Lindskog et al., 2017; Moscoso et al., 2020), with higher MA related to lower ANS task performance. However, other work has not shown this relationship (Dietrich et al., 2015; Wang et al., 2015; Hart et al., 2016; Braham and Libertus, 2018; Colomé, 2019; Szczygieł, 2021; Silver et al., 2022). Relatedly, research using non-symbolic tasks that are not related to ANS (e.g., comparing ratios of line segments) has also shown that MA does not relate as strongly to non-symbolic numerical tasks as it does to symbolic ones (Starling-Alves et al., 2022; Mielicki et al., 2023). What explains these mixed findings?

Although the negative relationship between MA and math performance is well-documented, there are different, though not mutually exclusive (Ashcraft, 2019), accounts of the direction of this relationship. Perhaps the most well-studied is the Disruption Account, which posits that math anxiety leads to anxious ruminations which capture working memory resources necessary for successfully completing a given math task (Eysenck and Calvo, 1992; see also Eysenck, 1997, 2013; Hopko et al., 1998; Ashcraft and Kirk, 2001; Beilock and DeCaro, 2007; Lee and Cho, 2018). Based on this account, completing an ANS task in a passive way (without active control) might lead to a stronger relationship between MA and ANS performance if it requires more working memory resources than completing an ANS task with active control. Another account, the Reduced Competency Account, posits that MA relates to, and possibly results from, a deficiency of basic as well as advanced math skills (Maloney et al., 2010; see also: Maloney et al., 2011; Maloney and Beilock, 2012; Núñez-Peña and Suárez-Pellicioni, 2014). Since the core sense of number supported by the ANS has been shown to relate to symbolic math skills (Libertus et al., 2011; Halberda et al., 2012; van Marle et al., 2018), the Reduced Competency Account might predict a negative relationship between MA and ANS task performance regardless of whether the ANS task is completed with active control. Finally, the Interpretation Account proposes that an individual’s appraisal of previous math experiences serve as indicators of lack of math ability, and that these appraisals lead to MA (Ramirez et al., 2018; see also Meece et al., 1990; Park et al., 2014). This account goes a step further than the reduced competency account to explain why not all those with low math ability develop MA and not all those with MA demonstrate low math ability – it is not the lower math ability in and of itself that leads to MA but rather an individual’s appraisal of perceived failure in math as an indicator of their own lack of math ability. According to this account, it might be unlikely that the ANS task should elicit MA since people generally do not have much experience with these tasks in typical math settings (i.e., math classes).

An emerging body of research has been exploring the link between MA and metacognitive processes in math. Metacognitive processes are those responsible for monitoring and controlling performance on cognitive tasks (Nelson and Narens, 1990; Ackerman and Thompson, 2017). Some work has indeed shown a relationship between MA and metacognitive processes in math. MA has been shown to negatively relate to confidence judgments in basic arithmetic tasks (Desender and Sasanguie, 2022) and in health-related math contexts (Rolison et al., 2016). MA may also be negatively related to metacognitive monitoring (Bellon et al., 2021), which is the extent to which judgments about one’s performance (e.g., “how confident are you that you answered correctly?”) align with actual performance on a math task. Finally, MA may negatively relate to cognitive reflection (Morsanyi et al., 2014). These findings suggest that presenting a numerical task in a way that encourages more metacognitive control could alter the relationship between MA and ANS.

Additionally, prior work suggests an interplay between MA, metacognitive processes and gender. On number line estimation tasks, males report higher item-level confidence than females even when controlling for actual performance (Rivers et al., 2021). A related line of work has also shown that males report higher item-level confidence on health-related math problems than non-males, though this effect may be mediated by gender differences in MA (Scheibe et al., 2022). Although this work suggests gender differences in math-related metacognition, this possibility remains underexplored with non-symbolic tasks relating to core number sense.

### Current study

One goal of the current study was to better understand the relationship between ANS task performance, metacognitive processes related to performance, and affective factors, specifically MA. Given the mixed evidence for a relationship between MA and ANS task performance, we tested whether giving participants active control in completing a numerical task would change the relationship between MA and ANS performance. In this case, we would expect to observe a negative relationship between MA and ANS performance for a passive ANS task, but this relationship would be weakened or not present in an active ANS task.

A second goal of this study was to test how MA and gender would relate to metacognitive processes during completion of ANS tasks. We measured metacognitive processes in two ways in the current study. In the passive ANS task, participants provided item-level confidence judgments, which reflect participants’ assessments of their own performance. In the active ANS task, participants had the opportunity to engage in active information seeking while completing the ANS task. We explored whether MA, gender, and other factors would predict confidence judgments or information seeking.

## Methods

### Participants

A sample of undergraduate students (*N* = 197) was recruited from the university subject pool, and students received course credit in exchange for participation. Participants were excluded based on preregistered^1^ criteria. First, participants who failed either of the two attention check questions embedded in the individual differences survey (*n* = 15) were removed. Next, participants who performed below chance (< 30% on the ANS task, *n* = 2) were removed. No participants were excluded for missing MA data. Next, participants with missing gender data (*n* = 38) were removed, followed by participants missing standardized math data^2^ (*n* = 32). Both of these items were optional in the demographic survey, which was submitted as part of a subject pool prescreening survey administered by the department. Finally, participants with little variability in their confidence ratings (same value for 75% of trials or more, *n* = 7) were removed. This resulted in a final sample size of *N* = 103 participants. Of this sample, 50 participants self-identified as male, 52 as female, and 1 as gender non-conforming.

### Materials

#### Math anxiety

Participants completed the Abbreviated Math Anxiety Scale (AMAS, Hopko et al., 1998), which is a 9-item measure of math anxiety. Participants were asked to indicate which best described their feelings toward each scenario (e.g., “Taking an examination in a math course.”) on a scale ranging from 1 = Low Anxiety to 5 = High Anxiety. Reliability was good (Cronbach’s ɑ = 0.84), and the sum of all responses was calculated for each participant with higher values indicating greater math anxiety.

#### General anxiety

Participants completed a 5-item measure of general anxiety based on Spielberger et al. (1970). Participants saw the following prompt: “A number of statements which people have used to describe themselves are given below. Read each statement and then click the response that indicates HOW YOU GENERALLY FEEL. There are no right or wrong answers. Do not spend too much time on any one statement but give the answer which seems to describe how you generally feel.” After reading the prompt, participants indicated how often they felt the way five statements described (e.g., “Some unimportant thoughts run through my mind and bothers me.”), with response scales ranging from 1 = “not at all” to 4 = “very much so.” Reliability was acceptable (Cronbach’s ɑ = 0.78), and the sum of all responses was calculated for each participant with higher values indicating greater general anxiety.

#### Demographics

Participants self-reported their gender identification from the options: male, female, and other. If participants selected “other,” they were prompted to describe their gender orientation in a text box. Participants also reported their math SAT score (out of 800 possible), and this was included in the models below as a measure of general math ability.

#### Passive ANS task

**Figure 1:**
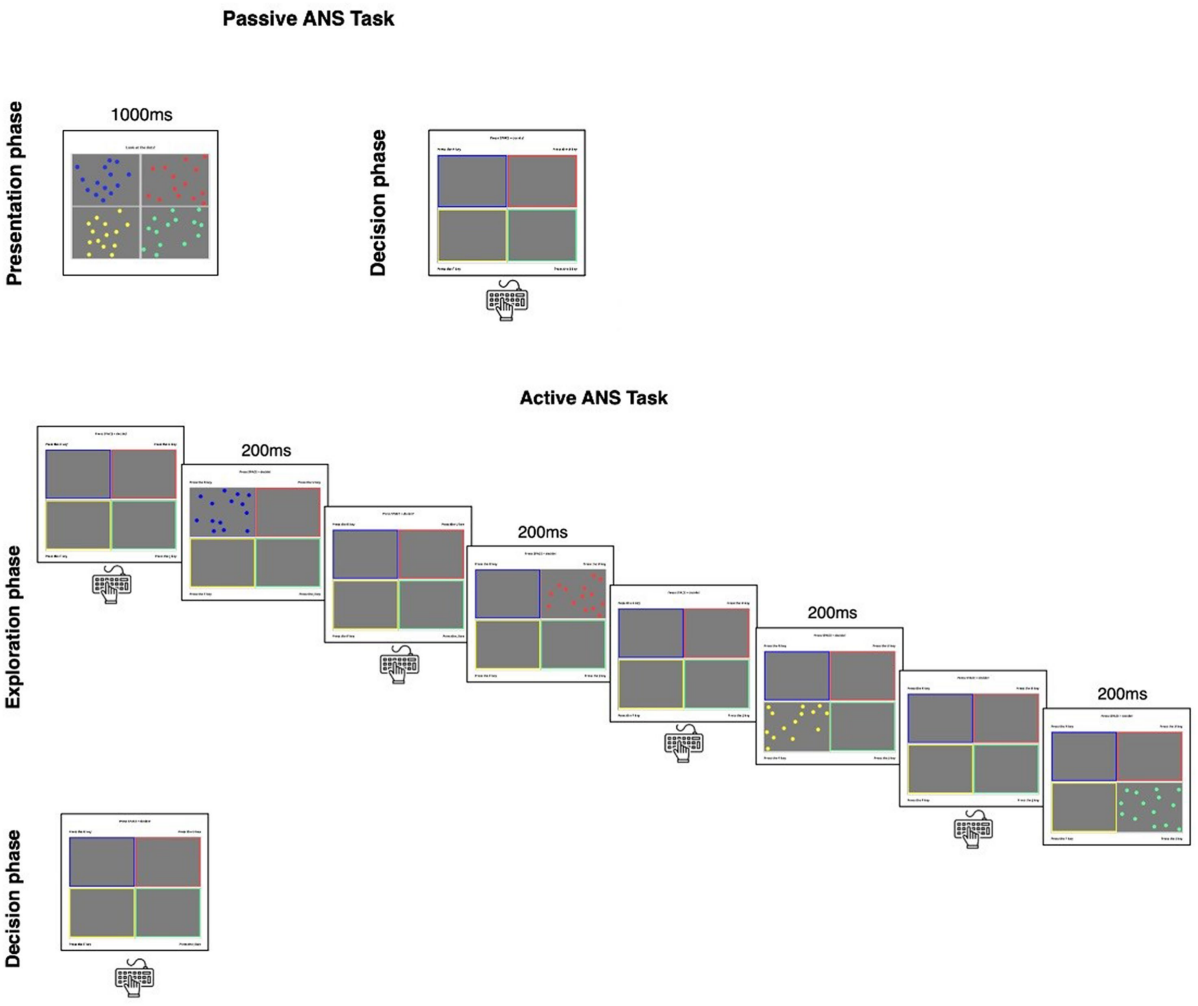
The non-symbolic numerical comparison task. During the passive ANS task (top panel), a grid with four arrays of dots was presented to participants for 1,000 ms. Then participants selected which box contained the largest quantity. During the active version of the task (bottom panel), participants did not see all four arrays at once. Instead, they pressed the corresponding key to view a single array for 200 ms. They could view each array as many times as they wished before indicating which box contained the largest quantity.

We used the same materials as Wang and Bonawitz (2019). For each trial, participants saw a 2×2 array for 1 s and were asked to indicate which of four arrays contained the most dots (see Figure 1). One of the arrays depicted a larger quantity than the other three, which were identical in quantity. Item difficulty was manipulated within-participants by changing the ratio (larger number/smaller number) between the arrays of dots for each trial (smaller ratio = higher difficulty, see Supplementary Table S1 for breakdown of items for each ratio). The difficulty levels were selected to match the trials in the Active ANS task (described in more details below). In order to maximize the opportunity to observe participants’ trade-off behavior during information seeking in the Active ANS task (i.e., seeking information more when trials were moderately difficult, and seeking information less when the trials were too easy or too difficult), trial difficulty ranged from extremely easy (i.e., a ratio of 2) to impossibly difficult (i.e., a ratio of 1). In addition, consistent with previous research (Wang and Bonawitz, 2019), we included more trials for the difficulty level close to the discrimination threshold for average adult participants (i.e., ratio ~ 1.1; Halberda and Feigenson, 2008). For the “impossibly difficult” trials (i.e., ratio of 1), the “correct” response was randomly pre-selected for the purpose of the task and the analyses, such that participants should perform at chance level (25%). Participants completed 50 trials without feedback, and accuracy was computed as proportion correct.

#### Confidence judgments

After each trial of the passive ANS task, participants indicated their confidence in their response by responding via a sliding scale to the following prompt: “How confident were you in your decision just now?.” The scale went from 0 to 10, with lower scores indicating lower confidence.

#### Active ANS task

The materials were the same as the active version of the task, but participants completed different trials than in the passive version. Participants were presented with a 2×2 grid (see bottom panel of Figure 1) and pressed one of four letter keys to view the corresponding array for 200 ms. Participants could view each array as many times as they wished before pressing the spacebar to make their selection of the largest quantity. Participants completed eight practice trials, and then as many trials as they could in 5 min. A progress bar remained on the screen as participants completed the task. Participants’ total score was displayed to discourage participants from idling (i.e., in theory, a participant could do nothing and wait for 5 min to pass). Accuracy was computed as proportion correct out of the total number of trials completed, which varied by participant (*M* = 56.17, *SD* = 23.40).

### Procedure

Participants completed the individual differences measures and the two versions of the ANS task during different sessions completed 1 to 2 days apart. During the first session, participants completed the math anxiety measure first, followed by the general anxiety measure and the demographic questionnaire. For both the math anxiety and general anxiety measures, all items were presented on the screen at the same time with item order randomized by participant. During the second session, participants completed the passive ANS task, followed by the active ANS task. During the passive version of the task, participants provided confidence judgments after completing each item.

## Results

### Overview of analyses

We used Cook’s distance to identify multivariate outliers in our data based on participants’ relationship between MA and ANS performance. If a participant had a Cook’s distance greater than 4/sample size for either the passive or the active version of the task, then they were excluded from analyses. For the passive task, data from two participants was excluded, and for the active task data from four participants was excluded resulting in a final sample size of *N* = 97. The correlations and descriptive statistics presented in Table 1 were computed with these outliers removed, but correlations and descriptive statistics for the full data set can be found in Supplementary Table S2. Consistent with prior findings (Else-Quest et al., 2010; Devine et al., 2012; Hart and Ganley, 2019), non-males in our sample reported higher MA (*M* = 28.90, *SD* = 5.49) than males (*M* = 25.50, *SD* = 5.80), *t*(95) = 3.00, *p* = 0.004, *d* = 0.60.

**Table 1:** Descriptive statistics and correlations with confidence intervals for possible ANS problems.

Variable	*M*	*SD*	1	2	3	4	5	6
1. MA (out of 45)	27.26	5.87						
2. ANS passive proportion correct	0.62	0.07	−0.24*					
			[−0.42, −0.04]					
3. ANS active proportion correct	0.67	0.08	0.06	0.18				
			[−0.14, 0.25]	[−0.02, 0.37]				
4. Confidence (out of 10)	6.36	1.58	−0.13	0.03	−0.27**			
			[−0.32, 0.07]	[−0.17, 0.23]	[−0.44, −0.07]			
5. Information Seeking	11.41	10.59	0.09	0.08	0.49**	−0.19		
			[−0.11, 0.28]	[−0.12, 0.28]	[0.32, 0.63]	[−0.38, 0.01]		
6. SAT math (out of 800)	675.73	85.24	−0.26*	0.04	−0.19	0.05	−0.21*	
			[−0.44, −0.06]	[−0.16, 0.24]	[−0.38, 0.01]	[−0.15, 0.25]	[−0.40, −0.02]	
7. General anxiety (out of 20)	14.56	3.54	0.43**	−0.16	0.15	−0.19	0.15	−0.11
			[0.25, 0.58]	[−0.35, 0.04]	[−0.05, 0.34]	[−0.38, 0.01]	[−0.05, 0.34]	[−0.30, 0.09]

M and SD are used to represent mean and standard deviation, respectively. Values in square brackets indicate the 95% confidence interval for each correlation. The confidence interval is a plausible range of population correlations that could have caused the sample correlation (Cumming, 2014). MA, math anxiety. Information seeking was the number of times participants chose to click through the cells in the 2×2 array. *Indicates *p* < 0.05. **Indicates *p* < 0.01.

All mixed-effects models were fit using the lme4 package (Bates et al., 2013) as in R (version 4.1.1; R Core Team, 2020). Models were fit using restricted maximum likelihood estimation (REML). When the outcome of interest was dichotomous (e.g., item-level accuracy), we fit logistic models, and when the outcome of interest was continuous (e.g., confidence), we fit linear models. For all mixed-effects models reported below, we followed an approach recommended by Barr (2013) to simplify the random-effects structure when necessary. We first ran each model with the maximal random structure, including random intercepts at the subject and item levels as well as subject-level random slopes. If the model failed to converge, we first fixed the correlation between slopes and intercepts to zero, then eliminated any random effects depending on which explained the least variance. We obtained *p* values using likelihood-ratio tests comparing the full model with the effect in question and the model without the effect in question. Parameters are evaluated with *t*-tests or *z*-tests (for individual contrasts) using Satterthwaite’s method for estimating degrees of freedom. To further evaluate contrasts and test the simple slopes for each level of the factor of interest, we used the emtrends function in emmeans (Lenth et al., 2020), which reports *t*-tests associated with individual contrasts with Satterthwaite’s method for estimating degrees of freedom.

For all models reported below, gender was recoded such that non-males (48 self-identified females and one self-identified gender-nonconforming participant in the final sample) were the reference group. Measures of general anxiety and participants’ self-reported standardized math scores were included as covariates in all models. All continuous predictors were rescaled for ease of interpretation, such that *M* = 0, *SD* = 1.

### ANS performance

**Figure 2:**
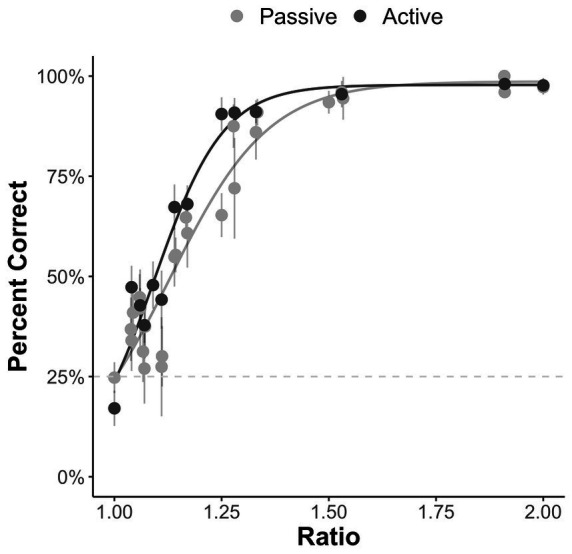
Percent correct for the passive and active ANS tasks by ratio. Error bars represent the 95% confidence interval around the mean.

We restricted our analysis to the subset of possible problems (ratio > 1). Descriptive statistics and correlations^3^ between measures are displayed in Table 1. As can be seen in Figure 2, we also replicated prior work (Barth et al., 2003; Piazza et al., 2004) showing a strong effect of ratio on accuracy in both passive and active versions of the task.

To test whether the relationship between MA and ANS performance varied depending on task type accounting for item difficulty, we fit a logistic mixed-effects model for item-level ANS accuracy with ratio, the task by MA interaction, all main effects, and covariates as fixed effects and subject-level ratio slopes as random effects. We also included the average of each participant’s item-level confidence ratings as a fixed factor in the model, to test whether MA would explain unique variance in the model. As shown in Table 2, this model also revealed a main effect MA, suggesting that, even accounting for item-level difficulty, higher MA was associated with lower item-level ANS accuracy. However, there was a significant task by MA interaction. Follow-up analyses indicated that there was a negative relation between MA and item-level ANS accuracy only for the passive ANS task.

**Table 2:** Logistic mixed-effect model for item-level ANS accuracy with task by MA interaction.

Fixed effects	OR	*b* (SE)	95% CI	ꭓ^2^	Random effects	Variance
Constant		−5.72 (0.48)	[−6.66, −4.79]	143.50^***^	Ratio|Subject	0.03
Task	1.13	0.12 (0.11)	[−0.10, 0.34]	1.20	Item (Intercept)	0.58
MA	0.88	−0.13 (0.05)	[−0.22, −0.03]	7.26^**^		
Ratio	235.55	5.46 (0.37)	[4.74, 6.19]	218.17^***^		
Confidence	0.97	−0.03 (0.02)	[−0.07, 0.01]	1.89		
Gender	0.95	−0.05 (0.07)	[−0.20, 0.10]	0.45		
SAT math	0.97	−0.03 (0.04)	[−0.10, 0.04]	0.75		
General anxiety	1.00	0.00 (0.04)	[−0.07, 0.08]	0.01		
Task*Math anxiety	1.15	0.14 (0.05)	[0.04, 0.24]	7.69^**^		

Non-males and the passive ANS task were the reference groups. MA, mean-centered math anxiety. AIC = 9,672, BIC = 9,750, Log Likelihood = −4,825. ^*^p < 0.05. ^**^p < 0.01. ^***^p < 0.001.

We also tested the possibility of a three-way interaction between task type, item difficulty (ratio), and math anxiety. We fit a logistic mixed-effects model for item-level accuracy which included the three-way interaction term, all lower-order interaction terms, main effects, and covariates as fixed effects and subject-level ratio slopes as random effects. As can be seen in Supplementary Table S3, the three-way interaction was not significant and adding the interaction term did not improve model fit, ꭓ^2^ = 0.00, *p* = 1.00, suggesting that the difference between passive and active versions of the task in the relation between MA and item-level ANS accuracy is similar at different levels of item difficulty.

To test whether gender moderated the difference in the MA-performance relation by task type, we fit a logistic mixed effects model with a task*MA*gender interaction term. As can be seen in Supplementary Table S4, there was no evidence of a three-way interaction between gender, task, and adding the interaction term did not improve model fit, ꭓ^2^ = 0.00, *p* = 1.00. This suggests that the different patterns of relations between MA and performance by task type did not vary by gender.

To address the possibility of order effects driving this pattern of results, we ran an additional analysis testing whether the relationship between MA and performance on the passive version of the task changed between the first and second half of the task. We fit a logistic mixed effects model with a timing*MA interaction term, all main effects, and covariates as fixed effects and item-level intercepts as random effects. As can be seen in Supplementary Table S7, suggesting a similar relationship between MA and passive ANS task performance during the first and second half of the task. These results do not support the possibility that practice with the ANS task is sufficient to change the relationship between MA and performance.^4^

Because of the self-controlled nature of the active ANS task, participants completed different numbers of trials from the passive ANS task. To ask whether the differences in trials between the two tasks drove the effects observed above, we also ran an additional analysis to explore the possibility that the different number of items completed by participants in the passive and active versions of the task could explain the different patterns observed for the relationship between MA and performance. We analyzed only the trials that participants completed in both the passive and active versions of the task and fit the same models described above: one model testing the two-way interaction between MA and task, one testing the three-way interaction between MA, task, and ratio, and one testing the three-way interaction between MA, task, and gender. The details of these analyses can be found in the Supplementary Tables S8–S10, but the pattern of results did not change when only this subset of items was analyzed. This does not support the possibility that the different items in each version of the task were driving the difference in the observed relationship between MA and performance (see Footnote 5).

Although there have been mixed arguments about whether accuracy or Weber fractions should be used to better estimate participants’ true ANS capacity (e.g., Lindskog et al., 2013; Inglis and Gilmore, 2014), we estimated Weber fractions (see Footnote 5) for each participant in the active and passive task by fitting participant data to a sigmoid model. Since there is no established model for the current 4-alternative-forced-choice ANS paradigm, we fit each participants’ data with a self-starting non-linear logistic model using the SSlogis function in R. This approach is similar to previous attempts to model the classic 2-alternative forced-choice ANS paradigm using sigmoid models, which provides best fits for participants’ data in order to estimate Weber fractions (Pica et al., 2004; Halberda and Feigenson, 2008). The final sample of participants with interpretable Weber fraction was *N* = 85 (models failed to fit for one participant in the passive task and 11 participants for the active task). We then regressed the Weber fractions onto MA including gender, standardized math, and general anxiety as covariates. The details of these analyses can be found in the Supplementary Tables S11–S13, but overall, there was no significant relationship between MA and Weber fraction for either the passive or active task. However, these results should be interpreted with caution due to low reliability of Weber fractions in both tasks,^5^ which is not unexpected given that both tasks in the current study had fewer than 300 trials (Lindskog et al., 2013).

### Confidence judgments

We tested whether MA or gender would emerge as significant predictors for item-level confidence judgments when accounting for item difficulty. We fit a linear mixed effects model with ratio, MA, gender, passive ANS performance, and covariates as fixed effects, and subject-and item-level intercepts as random effects. As can be seen in Table 3, ratio and gender emerged as significant predictors of item-level confidence judgments. Participants reported higher confidence for easier relative to more difficult items, and males reported higher item-level confidence judgments than non-males. We also ran a linear mixed-effects model for item-level confidence judgments with a MA by Gender by Ratio interaction term, but neither the three-way interaction nor any of the lower-order interactions reached significance (see Supplementary Table S5).

**Table 3:** Linear mixed-effects model for item-level confidence judgments.

Fixed effects	*b* (SE)	95%CI	*t*-value	df	Random effects	Variance
Constant	1.28 (1.45)	[−1.56, 4.11]	0.88	107.31	Subject (Intercept)	2.37
Ratio	3.93 (0.34)	[3.26, 4.60]	11.54^***^	48.00	Item (Intercept)	0.38
MA	−0.01 (0.19)	[−0.37, 0.36]	−0.05	91.00		
Gender	0.81 (0.35)	[0.12, 1.49]	2.32^*^	91.00		
ANS Passive Performance	−0.22 (2.20)	[−4.54, 4.09]	−0.10	91.00		
SAT math	−0.10 (0.17)	[−0.44, 0.24]	−0.57	91.00		
General Anxiety	−0.25 (0.18)	[−0.60, 0.009]	−1.45	91.00		

Non-males were the reference group. MA, mean-centered math anxiety. ^*^p < 0.05. ^***^p < 0.001.

### Information seeking

For these analyses, we examined information seeking at the item level, operationalized as the number of times participants chose to click through the arrays for each item.^6^ First, we tested whether engaging in information seeking improved ANS performance on possible items in the active version of the task similarly for all levels of item difficulty. We fit a logistic mixed-effects model for item-level ANS accuracy with the ratio by information seeking interaction and all main effects as fixed effects and subject-level and item-level intercepts as random effects. The information seeking by ratio interaction did not reach significance, *b* = 0.05, *SE* = 0.03, 95% CI[−0.02, 0.11], *p* = 0.151, and adding the interaction term did not improve model fit, ꭓ^2^ = 1.67, *p* = 0.200. This suggests that information seeking was beneficial for possible items at all levels of difficulty.

We also tested whether gender or MA moderate the relationship between information seeking and item-level ANS accuracy accounting for item difficulty.^7^ We fit a logistic mixed-effects model for item-level ANS accuracy with the information seeking by MA by gender interaction, all lower-order interactions, all main effects, and ratio as fixed effects and subject-level and item-level intercepts as random effects. As can be seen in Table 4, the three-way interaction of information seeking by MA by gender did not reach significance. However, there was a significant two-way interaction of information seeking by gender, and follow-up analyses suggest that males benefited more from information seeking than non-males.

**Table 4:** Logistic mixed-effects model for item-level ANS accuracy with information seeking by MA by gender interaction.

Fixed effects	OR	*b* (SE)	95% CI	ꭓ^2^	Random effects	Variance
Constant		−5.85 (0.55)	[−6.92, −4.78]	114.55^***^	Subject (Intercept)	0.04
Information seeking	1.00	0.00 (0.01)	[−0.01, 0.01]	0.02	Item (Intercept)	0.50
MA	0.96	−0.04 (0.07)	[−0.17, 0.09]	0.34		
Gender	0.82	−0.20 (0.09)	[−0.37, −0.03]	5.42^*^		
Ratio	264.13	5.58 (0.45)	[4.70, 6.46]	154.16^***^		
Info. Seeking*MA	1.00	0.00 (0.01)	[−0.01, 0.01]	0.09		
Info. Seeking*Gender	1.03	0.03 (0.01)	[0.01, 0.04]	7.82^**^		
MA*Gender	1.07	0.07 (0.09)	[−0.10, 0.24]	0.59		
Info. Seeking*MA* Gender	1.00	0.00 (0.01)	[−0.02, 0.02]	0.08		

Non-males were the reference group. MA, mean-centered math anxiety. AIC = 5,182, BIC = 5,254, Log Likelihood = −2,580. ^*^p < 0.05. ^**^p < 0.01. ^***^p < 0.001.

Finally, we tested whether any significant predictors emerged for item-level information seeking when accounting for item difficulty. We fit a linear mixed effects model with ratio, MA, gender and covariates as fixed effects, and subject-and item-level intercepts as random effects. As can be seen in Table 5, only ratio emerged as a significant predictor of information seeking, with participants engaging in less information seeking for easier relative to more difficult items.

**Table 5:** Linear mixed-effects model for item-level information seeking.

Fixed effects	Estimate (*SE*)	95%CI	*t*-value	*df*	Random effects	Variance
Constant	14.41 (9.81)	[−4.83, 33.64]	1.47	86.96	Subject (Intercept)	96.75
Ratio	−4.90 (0.74)	[−6.35, −3.44]	−6.58^***^	108.68	Item (Intercept)	2.69
MA	−0.22 (1.19)	[−2.56, 2.12]	−0.18	85.45		
Gender	−1.37 (2.30)	[−5.88, 3.14]	−0.60	85.42		
Confidence	−0.96 (0.67)	[−2.28, 0.35]	−1.43	85.20		
ANS passive performance	15.51 (14.14)	[−12.19, 43.22]	1.10	85.54		
SAT math	−1.77 (1.12)	[−3.96, 0.41]	−1.59	85.37		
General anxiety	1.23 (1.14)	[−0.99, 3.46]	1.09	85.50		

Non-males were the reference group. MA, mean-centered math anxiety. ^***^p < 0.001.

## Discussion

We set out to test whether giving participants active control during an ANS task would change the relationship between MA and task performance. We also explored the relationships between MA, ANS task performance, gender, and metacognitive processes. Participants completed a passive version of a novel four-alternative-forced-choice non-symbolic numerical comparison task followed by an active version in which they had the opportunity to engage in additional information seeking before responding. During the passive version of the task, participants also provided a confidence judgment after each item.

First, we found that participants’ performance on the novel four-alternative-forced-choice non-symbolic numerical comparison task follows the ratio-dependent signature of the ANS, that is, participants performed better on the task when the ratio between the quantities was larger. Interestingly, this was true for both the active and passive versions of the task. In the active version of the task, participants had the opportunity to view the dot arrays as many times as they wanted before making a decision. Despite this, participants’ performance was still ratio dependent. Interestingly, we did not find any significant difference in participants’ performance between the passive and active ANS tasks when accounting for the difficulty of the numerical comparisons, although individual differences in information seeking predicted participants’ performance on the active version of the task. Previous research suggests that numerical decisions are based on serially collected cumulative information (Cheyette and Piantadosi, 2019). Our results provide converging evidence that information seeking benefits ANS performance. However, the lack of item-level difference between the active and passive version of the task when controlling for trial difficulty suggests that there is a limit to how much additional information improves ANS performance – and this limit may be very close to the participants’ existing discrimination threshold. However, the current study was not specifically designed to test the cognitive mechanism of the ANS. Future work should systematically examine the influence of having additional information, either passively or actively, on ANS performance.

Next, we replicated prior work (Lindskog et al., 2017; Moscoso et al., 2020) showing that ANS accuracy negatively correlates with MA during the passive task. Critically, however, there was a significant interaction between MA and task type on item-level accuracy, suggesting that the opportunity to engage in active information seeking moderated the relationship between MA and ANS task performance, and that active task administration seemed to attenuate this relationship. These findings are not consistent with the Reduced Competency Account (Maloney et al., 2010), which would predict a negative relationship between MA and ANS tasks regardless of passive or active administration since both tasks tap basic number sense which may be weaker in people with higher MA. These findings are also not consistent with the Interpretation Account (Ramirez et al., 2018), which would not predict a relationship between MA and ANS task performance since participants are unlikely to have specific negative experiences with the ANS task that they appraise as reflecting poorly on them as math learners. The current findings are most in line with the Disruption Account (Eysenck and Calvo, 1992), assuming that the passive version of the ANS task in the current study relied more heavily on working memory resources than the active ANS task. The additional finding that MA did not relate to participants’ Weber fractions for either task, and the finding that accuracy on both tasks followed a typical ratio-dependent signature, also supports the possibility that MA may impact participants’ overall attention level as opposed to ANS acuity. Again, however, this finding cannot be given too much weight due to the low reliability of the Weber fractions in both tasks.

Relatedly, all four arrays of dots were shown to participants simultaneously in the passive but not active version of the task (see Footnote 5). This difference between tasks relates to the possible role of working memory capacity in the relationship between MA and performance on mathematical tasks (disruption account). If viewing all four arrays at the same time is more taxing for working memory, and if MA consumes additional working memory resources which cannot then be allocated toward task performance, this could lead to lower performance. However, in order to speak directly to the potential role of working memory in these findings, future work should include measures of working memory capacity and examine its role in mediating the link between ANS and MA.

Participants were sensitive to the task difficulty when deciding to seek additional information during the active task, as evidenced by the significant effect of ratio on information seeking. This suggests that there may be social–emotional motivation to seek information in the ANS task that goes beyond direct performance boost. The interaction between gender and information seeking tentatively supports this interpretation, suggesting that engaging in information seeking benefitted males more than non-males. Our findings also contribute to other work showing that males and non-males differ in the extent to which they report experiencing MA (Else-Quest et al., 2010; Devine et al., 2012; Hart and Ganley, 2019), and in their confidence on numerical tasks (Rivers et al., 2021; Scheibe et al., 2022). Future work should continue to explore other social–emotional predictors that could shed light on the relationships between numerical task performance, gender, and metacognitive processes.

Interestingly, we did not find significant links between participants’ self-reported confidence level and their performance or information seeking, although confidence ratings were also ratio dependent. This may be because explicit reports of confidence tap into different underlying processes from the implicit confidence levels that underlie participants’ performance or information seeking. It is less likely, although possible, that participants’ confidence levels are entirely separated from their performance and information seeking. Alternatively, it is also possible that the self-reported measures were not sensitive enough to subtle variations in participants’ internal confidence. Future research should use different types of confidence measures, such as having participants wager on their decisions (e.g., Vo et al., 2014), to further investigate the relationships between confidence, performance, and MA.

Although the interaction between MA and task type on item-level accuracy supports the possibility that the active or passive administration of the task changes the relationship between MA and task performance, there are other potential alternative explanations for the findings. First, it is possible that the order of tasks in the current study drove the difference in relationship between MA and the passive vs. active versions of ANS tasks since the passive ANS task always preceded the active ANS task. However, if the difference was entirely driven by temporal order of task administration, we would expect differences in the relationship between MA and the first vs. second half of the passive ANS task. This was not found in our additional analyses. Furthermore, other work (Conlon et al., 2021) has found that the relationship between MA and performance does not change when MA is measured at different timepoints (before, during, and after a math fluency task).

Second, it is possible that the difference in the number of items that participants experienced in the passive and active ANS tasks drove the difference in relationship between MA and the ANS tasks. However, even when we only analyzed the subset of items that were shared between the passive and active ANS tasks, we found no difference in the pattern of observed results.

In addition to having different numbers of items, the active and passive versions of the task also differed in a number of ways, which could have implications for the relationship between MA and task performance. In the active version of the task participants were limited to 5 min for task completion, whereas participants could complete the passive task at their own pace. Evidence for the relationship between MA and performance on timed vs. untimed tasks is mixed (Caviola et al., 2017). However, some research has shown that time pressure can lead to lower performance on arithmetic tasks (Beilock and Carr, 2005) perhaps due to additional strain on working memory resources. This prior work would suggest a stronger negative relationship between MA and task performance on the active version of the task relative to the passive version since the active version was timed, which is not what was observed in the current study.

Another difference between the two versions of the ANS task was that a participant’s total score was shown on the screen during the trials in the active version of the task but not for the passive version. However, showing participants that they were not doing well during the active version of the task could potentially *increase* the strength of the negative relationship between MA and task performance, whereas we observed no evidence for any link between MA and active ANS performance in the current study.

Participants provided item-level confidence judgments during the passive version of the task, but not during the active version. Although we observed no correlation between MA and confidence judgments, it remains possible that explicit reflection on item-level confidence increased the link between MA and ANS performance, although previous research without such confidence measures also observed similar links between MA and ANS performance (Lindskog et al., 2017). Future research is needed to further investigate the links between self-reflection, cognitive control, task performance and anxiety.

We set out to ask whether giving participants active control over their numerical performance might alleviate some of the pressure from performing a numerical task, and consequently break the link between math anxiety and math performance. The current study focused on arguably the most “basic” aspect of math performance – our intuitive number sense. We found promising evidence that having active control attenuates the correlation between math anxiety and numerical accuracy – at least at the basic, number sense level. It remains to be tested how the kind of active control we used in the current study directly influences math anxiety and symbolic math performance.

## Data availability statement

Data and analytic code are available on the Open Science Framework (page) for the project: https://osf.io/ye96s/.

## Ethics statement

The studies involving humans were approved by Rutgers University Institutional Review Board. The studies were conducted in accordance with the local legislation and institutional requirements. The participants provided their written informed consent to participate in this study.

## Author contributions

MM, RM, and JW contributed to conception and design of the study. RM coordinated data collection and assisted with programming. MM performed the statistical analysis and wrote the first draft of the manuscript. JW contributed to manuscript revision. All authors read and approved the submitted version.

## Footnotes

^1^This preregistration includes additional measures that were not analyzed as part of the current study. https://osf.io/h7twd

^2^Keeping these 32 participants in the data set does not change the pattern of results. See Supplementary Tables S14–S19 for additional analyses with these excluded participants.

^3^Split-half reliability estimates for ANS accuracy with Spearman-Brown corrections were calculated for possible problems using 5,000 random splits with the *splithalf* R package (Parsons, 2021). The reliability estimate for all possible items was 0.05, 95% CI [−0.02, 0.29] for passive and 0.22, 95% CI [−0.04, 0.44] for active. When only equated items were considered (i.e., the same number of items completed for active and passive by participant), the reliability estimate was −0.08, 95% CI [−0.32, 0.20] for passive and 0.13, 95% CI [−0.12, 0.37] for active. These results suggest that our current results may be underestimating the correlation between passive ANS and MA, more so than the correlation between active ANS and MA, suggesting that the true correlation between passive ANS and MA should be higher than the correlation between active ANS and MA.

^4^We are grateful to reviewers for this suggestion.

^5^We calculated reliability for the Weber fractions by fitting participant data to a sigmoid model separately for the first and second half of trials in the passive and active tasks. For the passive task, only *n* = 59 participants had successful model fit, and reliability was low, (*r* = 0.20, *p* = 0.100). For the active task, only *n* = 45 participants had successful model fit, and reliability was low, (*r* = 0.02, *p* = 0.900).

^6^We also tested whether information seeking impacted item-level accuracy on the active ANS task accounting for item difficulty. As can be seen in Supplementary Table S6, both ratio and information seeking significantly predicted item-level accuracy on possible problems in the active ANS task.

^7^We also ran a model with an information seeking by gender by MA by ratio four-way interaction term, but this model failed to converge.
