# Supplementary material for: Understanding the social–emotional components of our “number sense”: insights from a novel non-symbolic numerical comparison task
Source: Front Psychol. 2024 Mar 5;15:1175591. doi: 10.3389/fpsyg.2024.1175591 (PMC10948494; doi:10.3389/fpsyg.2024.1175591)
Supplement: Supplementary file 1 [file Data_Sheet_1.docx]

**Supplemental Materials**

**Table S1**

*Number of Items by Ratio in Passive ANS Task*

| Ratio | Items | Number of Dots per Array for each Item |
| --- | --- | --- |
| 1 | 5 | 14:14, 14:14, 21:21, 21:21, 21:21 |
| 1.04 | 6 | 23:24, 23:24, 23:24, 23:24, 26:27, 26:27 |
| 1.06 | 6 | 15:16, 15:16, 17:18, 17:18, 17:18, 17:18 |
| 1.1 | 4 | 9:10, 9:10, 14:15, 14:15 |
| 1.15 | 12 | 12:14, 12:14, 14:16, 14:16, 14:16, 18:21, 18:21, 18:21, 21:24, 21:24, 21:24, 21:24 |
| 1.25 | 5 | 12:15, 12:15, 12:15, 18:23, 18:23 |
| 1.33 | 4 | 12:16, 12:16, 18:24, 18:24 |
| 1.5 | 4 | 10:15, 10:15, 15:23, 15:23 |
| 2 | 4 | 7:14, 7:14, 7:14, 11:21 |

*Note.* For each trial, there were 3 arrays with the same number of dots and one array (the correct answer) with a different number of dots.

**Table S2**

*Descriptive Statistics and Correlations with Confidence Intervals for Possible ANS Problems for Full Sample*

| Variable | *M* | *SD* | 1 | 2 | 3 | 4 | 5 | 6 |
| --- | --- | --- | --- | --- | --- | --- | --- | --- |
| 1. MA (out of 45) | 27.36 | 6.20 |  |  |  |  |  |  |
| 2. ANS Passive Proportion Correct | 0.62 | 0.08 | -.23^*^ |  |  |  |  |  |
|  |  |  | [-.41, -.04] |  |  |  |  |  |
| 3. ANS Active Proportion Correct | 0.67 | 0.09 | .03 | .21^*^ |  |  |  |  |
|  |  |  | [-.16, .24] | [.02, .39] |  |  |  |  |
| 4. Confidence (out of 10) | 6.36 | 1.59 | -.19 | .00 | -.21^*^ |  |  |  |
|  |  |  | [-.37, .00] | [-.19, .20] | [-.39, -.02] |  |  |  |
| 5. Information Seeking | 11.66 | 11.62 | .11 | .12 | .53^**^ | -.17 |  |  |
|  |  |  | [-.08, .30] | [-.07, .31] | [.38, .66] | [-.35, .02] |  |  |
| 6. SAT Math (out of 800) | 671.35 | 88.09 | -.27^**^ | .06 | -.07 | .06 | -.22^*^ |  |
|  |  |  | [-.44, -.08] | [-.14 .25] | [-.26, .13] | [-.13, .25] | [-.40, -.03] |  |
| 7. General Anxiety (out of 20) | 14.64 | 3.50 | .43^**^ | -.14 | .10 | -.20^*^ | .17 | -.16 |
|  |  |  | [.25, .57] | [-.32, .06] | [-.09, .29] | [-.38, -.01] | [-.03, .35] | [-.34, .04] |

*Note.* *M* and *SD* are used to represent mean and standard deviation, respectively. Values in square brackets indicate the 95% confidence interval for each correlation. The confidence interval is a plausible range of population correlations that could have caused the sample correlation (Cumming, 2014). MA = math anxiety. Information seeking was the number of times participants chose to click through the cells in the 2x2 array.

^*^ indicates *p* < .05. ^**^ indicates *p* < .01.

**Table S3**

*Logistic Mixed-Effect Model for Item-Level ANS Accuracy with Task by MA by Ratio Interaction*

| Fixed Effects |  |  |  |  | Random Effects |  |
| --- | --- | --- | --- | --- | --- | --- |
|  | OR | *b* (SE) | 95% CI | ꭓ^2^ |  | Variance |
| Constant |  | -5.67 (0.65) | [-6.95, -4.38] | 78.04^***^ | Item (Intercept) | 0.57 |
| Task | 1.02 | 0.02 (0.75) | [-1.46, 1.50] | 0.00 |  |  |
| MA | 1.76 | 0.57 (0.33) | [-0.08, 1.21] | 2.98 |  |  |
| Ratio | 224.98 | 5.42 (0.54) | [4.36, 6.47] | 101.65^***^ |  |  |
| Confidence | 0.97 | -0.03 (0.02) | [-0.06, 0.00] | 3.10 |  |  |
| Gender | 0.95 | -0.06 (0.06) | [-0.17, 0.06] | 0.93 |  |  |
| SAT Math | 0.97 | -0.03 (0.03) | [-0.08, 0.03] | 0.98 |  |  |
| General Anxiety | 1.00 | 0.00 (0.03) | [-0.05, 0.06] | 0.00 |  |  |
| Task*Math Anxiety | 0.60 | -0.52 (0.44) | [-1.39, 0.35] | 1.37 |  |  |
| Task*Ratio | 1.08 | 0.07 (0.64) | [-1.18, 1.32] | 0.01 |  |  |
| Math Anxiety*Ratio | 0.55 | -0.60 (0.28) | [-1.16, 0.05] | 4.54^*^ |  |  |
| Task*Math Anxiety*Ratio | 1.78 | 0.57 (0.38) | [-0.18, 1.32] | 2.24 |  |  |

*Note.* Non-males and the passive ANS task were the reference groups. MA = mean-centered math anxiety. AIC = 9687, BIC = 9780, Log Likelihood = -4830.

^*^ indicates *p* < .05, ^***^ indicates *p* < .001.

**Table S4**

*Logistic Mixed-Effect Model for Item-Level ANS Accuracy with Task by MA by Gender Interaction*

| Fixed Effects |  |  |  |  | Random Effects |  |
| --- | --- | --- | --- | --- | --- | --- |
|  | OR | *b* (SE) | 95% CI | ꭓ^2^ |  | Variance |
| Constant |  | -5.72 (0.47) | [-6.63, -4.80] | 148.85^***^ | Item (Intercept) | 0.57 |
| Task | 1.25 | 0.23 (0.13) | [-0.02, 0.47] | 3.27 |  |  |
| MA | 0.89 | -0.12 (0.06) | [-0.23, -0.01] | 4.20^*^ |  |  |
| Ratio | 221.34 | 5.40 (0.37) | [4.68, 6.12] | 215.47^***^ |  |  |
| Confidence | 0.97 | -0.03 (0.02) | [-0.06, 0.00] | 2.83 |  |  |
| Gender | 1.04 | 0.04 (0.08) | [-0.12, 0.20] | 0.27 |  |  |
| SAT Math | 0.97 | -0.03 (0.03) | [-0.08, 0.03] | 1.05 |  |  |
| General Anxiety | 1.00 | 0.00 (0.03) | [-0.06, 0.06] | 0.00 |  |  |
| Task*Math Anxiety | 1.09 | 0.09 (0.08) | [-0.07, 0.25] | 1.26 |  |  |
| Task*Gender | 0.83 | -0.19 (0.11) | [-0.40, 0.02] | 3.11 |  |  |
| Math Anxiety*Gender | 1.01 | 0.01 (0.08) | [-0.15, 0.16] | 0.01 |  |  |
| Task*Math Anxiety*Gender | 1.04 | 0.04 (0.11) | [-0.17, 0.25] | 0.16 |  |  |

*Note.* Non-males and the passive ANS task were the reference groups. MA = mean-centered math anxiety. AIC = 9688, BIC = 9781, Log Likelihood = -4831.

^*^ indicates *p* < .05, ^***^ indicates *p* < .001.

**Table S5**

*Linear Mixed-Effects Model for Item-Level Confidence Judgments with MA by Gender by Ratio Interaction*

| Fixed Effects |  |  |  |  | Random Effects |  |
| --- | --- | --- | --- | --- | --- | --- |
|  | *b* (*SE*) | 95%CI | *t*-value | *df* |  | Variance |
| Constant | 1.01 (1.46) | [-1.85, 3.87] | 0.69 | 107.94 | Subject (Intercept) | 2.33 |
| Ratio | 4.11 (0.36) | [3.40, 4.81] | 11.44^***^ | 59.24 | Item (Intercept) | 0.37 |
| MA | 0.44 (0.32) | [-0.18, 1.06] | 1.38 | 233.92 |  |  |
| Gender | 1.16 (0.44) | [0.29, 2.03] | 2.62^*^ | 226.79 |  |  |
| ANS Passive Performance | -0.19 (2.21) | [-4.52, 4.15] | -0.08 | 90.00 |  |  |
| SAT Math | -0.10 (0.17) | [-0.44, 0.24] | -0.57 | 90.00 |  |  |
| General Anxiety | -0.24 (0.18) | [-0.59, 0.11] | -1.34 | 90.00 |  |  |
| MA*Ratio | -0.28 (0.16) | [-0.60, 0.03] | -1.78 | 4701.00 |  |  |
| Gender*Ratio | -0.22 (0.22) | [-0.71, 0.15] | -1.29 | 4701.00 |  |  |
| MA*Gender | -0.50 (0.43) | [-1.35, 0.34] | -1.17 | 244.44 |  |  |
| MA*Gender*Ratio | 0.25 (0.22) | [-0.18, 0.68] | 1.13 | 4701.00 |  |  |

Note*.* Non-males were the reference group. MA = mean-centered math anxiety.

^*^ indicates *p* < .05. ^***^ indicates *p* < .001.

**Table S6**

*Logistic Mixed-Effect Model for Item-Level Accuracy on Active Task Items*

| Fixed Effects |  |  |  |  | Random Effects |  |
| --- | --- | --- | --- | --- | --- | --- |
|  | OR | *b* (SE) | 95% CI | ꭓ^2^ |  | Variance |
| Constant | 0.00 | -6.23 (0.53) | [-7.27, -5.18] | 136.46^***^ | Subject (Intercept) | 0.05 |
| Information Seeking | 1.01 | -0.01 (0.00) | [0.00, 0.02] | 4.57^*^ | Item (Intercept) | 0.45 |
| Ratio | 322.94 | 5.78 (0.44) | [4.91, 6.64] | 172.03^***^ |  |  |

*Note.* AIC = 5176, BIC = 5208, Log Likelihood = -2583.

^*^ indicates *p* < .05. ^***^ indicates *p* < .001.

**Table S7**

*Logistic Mixed-Effect Model for Item-Level Passive ANS Accuracy with MA by Timing Interaction*

| Fixed Effects |  |  |  |  | Random Effects |  |
| --- | --- | --- | --- | --- | --- | --- |
|  | OR | *b* (SE) | 95% CI | ꭓ^2^ |  | Variance |
| Constant |  | -6.20 (0.75) | [-7.67, -4.73] | 68.05^***^ | Item (Intercept) | 0.64 |
| MA | 0.89 | -0.11 (0.05) | [-0.21, -0.003] | 4.06^*^ |  |  |
| Timing | 1.34 | 0.29 (0.26) | [-0.21, 0.79] | 1.28 |  |  |
| Gender | 1.01 | 0.01 (0.08) | [-0.15, 0.18] | 0.02 |  |  |
| Ratio | 252.53 | 5.53 (0.59) | [4.38, 6.69] | 87.96^***^ |  |  |
| Confidence | 1.00 | -0.00 (0.02) | [-0.05, 0.05] | 0.01 |  |  |
| SAT Math | 0.99 | -0.01 (0.04) | [-0.09, 0.07] | 0.10 |  |  |
| General Anxiety | 0.97 | -0.03 (0.04) | [-0.11, 0.05] | 0.62 |  |  |
| MA*Timing | 1.03 | 0.03 (0.07) | [-0.11, 0.18] | 0.18 |  |  |

*Note.* Non-males and the first half of the passive ANS task were the reference groups. MA = mean-centered math anxiety. AIC = 4508, BIC = 4572, Log Likelihood = -2244.

^*^ indicates *p* < .05. ^***^ indicates *p* < .001.

We replicated the main analyses with only the subset of trials that each participant completed in both the passive and active versions of the task. If a participant completed more than 50 trials in the active version (*n* = 57) these trials were not analyzed. If a participant completed fewer than 50 trials in the active version (*n* = 38) these were not analyzed.

**Table S8**

*Logistic Mixed-Effect Model for Item-Level ANS Accuracy with Task by MA Interaction*

| Fixed Effects |  |  |  |  | Random Effects |  |
| --- | --- | --- | --- | --- | --- | --- |
|  | OR | *b* (SE) | 95% CI | ꭓ^2^ |  | Variance |
| Constant |  | -5.61 (0.52) | [-6.63, -4.59] | 115.89^***^ | Ratio\|Subject | 0.03 |
| Task | 1.29 | 0.26 (0.18) | [-0.09, 0.61] | 2.05 | Item (Intercept) | 0.61 |
| MA | 0.88 | -0.12 (0.05) | [-0.22, -0.03] | 6.75^**^ |  |  |
| Ratio | 209.11 | 5.34 (0.40) | [4.56, 6.13] | 176.91^***^ |  |  |
| Confidence | 0.97 | -0.03 (0.02) | [-0.08, 0.01] | 2.33 |  |  |
| Gender | 0.94 | -0.06 (0.08) | [-0.21, 0.09] | 0.66 |  |  |
| SAT Math | 0.97 | -0.04 (0.04) | [-0.11, 0.04] | 0.90 |  |  |
| General Anxiety | 0.99 | -0.01 (0.04) | [-0.09, 0.06] | 0.14 |  |  |
| Task*Math Anxiety | 1.12 | 0.12 (0.06) | [0.01, 0.23] | 4.42^*^ |  |  |

*Note.* Non-males and the passive ANS task were the reference groups. MA = mean-centered math anxiety. AIC = 7863, BIC = 7940, Log Likelihood = -3921.

^*^ indicates *p* < .05. ^**^*p* < .01. ^***^ indicates *p* < .001.

**Table S9**

*Logistic Mixed-Effect Model for Item-Level ANS Accuracy with Task by MA by Ratio Interaction*

| Fixed Effects |  |  |  |  | Random Effects |  |
| --- | --- | --- | --- | --- | --- | --- |
|  | OR | *b* (SE) | 95% CI | ꭓ^2^ |  | Variance |
| Constant |  | -5.90 (0.74) | [-7.36, -4.45] | 63.34^***^ | Item (Intercept) | 0.59 |
| Task | 1.97 | 0.68 (0.99) | [-1.26, 2.62] | 0.47 |  |  |
| MA | 2.03 | 0.71 (0.34) | [0.04, 1.38] | 4.27^*^ |  |  |
| Ratio | 268.58 | 5.60 (0.60) | [4.42, 6.77] | 87.15^***^ |  |  |
| Confidence | 0.97 | -0.03 (0.02) | [-0.07, 0.00] | 3.56 |  |  |
| Gender | 0.94 | -0.06 (0.06) | [-0.18, 0.07] | 0.82 |  |  |
| SAT Math | 0.97 | -0.03 (0.03) | [-0.09, 0.03] | 1.07 |  |  |
| General Anxiety | 0.98 | -0.02 (0.03) | [-0.08, 0.05] | 0.24 |  |  |
| Task*Math Anxiety | 0.67 | -0.39 (0.49) | [-1.35, 0.56] | 0.65 |  |  |
| Task*Ratio | 0.70 | -0.36 (0.81) | [-1.95, 1.22] | 0.20 |  |  |
| MA*Ratio | 0.49 | -0.72 (0.29) | [-1.30, -0.14] | 5.99^*^ |  |  |
| Task*MA*Ratio | 1.55 | 0.44 (0.43) | [-0.40, 1.27] | 1.06 |  |  |

*Note.* Non-males and the passive ANS task were the reference groups. MA = mean-centered math anxiety. AIC = 7868, BIC = 7958, Log Likelihood = -3921.

^*^ indicates *p* < .05. ^**^ indicates *p* < .01. ^***^ indicates *p* < .001.

**Table S10**

*Logistic Mixed-Effect Model for Item-Level ANS Accuracy with Task by MA by Gender Interaction*

| Fixed Effects |  |  |  |  | Random Effects |  |
| --- | --- | --- | --- | --- | --- | --- |
|  | OR | *b* (SE) | 95% CI | ꭓ^2^ |  | Variance |
| Constant |  | -5.61 (0.51) | [-6.62, -4.61] | 119.18^***^ | Item (Intercept) | 0.60 |
| Task | 1.45 | 0.37 (0.19) | [0.00, 0.74] | 3.85^*^ |  |  |
| MA | 0.89 | -0.11 (0.06) | [-0.23, 0.01] | 3.33 |  |  |
| Ratio | 198.72 | 5.29 (0.40) | [4.51, 6.08] | 174.72^***^ |  |  |
| Confidence | 0.97 | -0.03 (0.02) | [-0.07, 0.00] | 3.00 |  |  |
| Gender | 1.03 | 0.03 (0.09) | [-0.14, 0.20] | 0.12 |  |  |
| SAT Math | 0.97 | -0.03 (0.03) | [-0.09, 0.03] | 1.12 |  |  |
| General Anxiety | 0.98 | -0.02 (0.03) | [-0.08, 0.04] | 0.40 |  |  |
| Task*Math Anxiety | 1.02 | 0.02 (0.09) | [-0.15, 0.19] | 0.04 |  |  |
| Task*Gender | 0.83 | -0.19 (0.12) | [-0.41, 0.04] | 2.52 |  |  |
| MA*Gender | 1.01 | 0.01 (0.08) | [-0.16, 0.17] | 0.00 |  |  |
| Task*MA*Gender | 1.14 | 0.13 (0.12) | [-0.10, 0.36] | 1.22 |  |  |

*Note.* Non-males and the passive ANS task were the reference groups. MA = mean-centered math anxiety. AIC = 7870, BIC = 7960, Log Likelihood = -3922.

^*^ indicates *p* < .05. ^**^ indicates *p* < .01. ^***^ indicates *p* < .001.

We calculated Weber fractions for each participant in the active and passive task by fitting participant data to a sigmoid model and taking the SD parameter minus 1. The final sample of participants with positive Weber fraction values was *N* = 85 (one participant had a negative Weber fraction for the passive task and four for the active task and we were unable to calculate Weber fractions for seven participants for the active task). We then regressed the Weber fractions onto MA including gender, standardized math, and general anxiety as covariates. We ran separate linear regression models for the passive task (Table S11) and the active task (Table S12) and a combined mixed-effects model accounting for subject-level random intercepts (Table S13).

**Table S11**

*Linear Regression Model for Weber Fractions in the Passive Task*

|  | *b* (*SE*) | 95%CI | *t*-value |
| --- | --- | --- | --- |
| Constant | 0.12 (0.01) | [0.10, 0.14] | 14.18^**^ |
| MA | 0.00 (0.01) | [-0.01, 0.01] | 0.36 |
| Gender | 0.01 (0.13) | [-0.02, 0.03] | 0.44 |
| SAT Math | -0.00 (0.01) | [-0.02, 0.01] | -0.39 |
| General Anxiety | 0.00 (0.01) | [-0.01, 0.01] | 0.37 |

*Note.* Non-males were the reference group. MA = mean-centered math anxiety. Residual SE = 0.05. Multiple R^2^ = 0.01, Adjusted R^2^ = -0.04, *F*(4, 80) = 0.18, *p* = .949.

^**^ indicates *p* < .01.

**Table S12**

*Linear Regression Model for Weber Fractions in the Active Task*

|  | *b* (*SE*) | 95%CI | *t*-value |
| --- | --- | --- | --- |
| Constant | 0.09 (0.01) | [0.08, 0.10] | 15.28^***^ |
| MA | 0.00 (0.00) | [-0.01, 0.01] | 0.19 |
| Gender | -0.00 (0.01) | [-0.02, 0.01] | -0.43 |
| SAT Math | 0.01 (0.00) | [0.00, 0.02] | 2.00^*^ |
| General Anxiety | 0.00 (0.00) | [-0.01, 0.01] | 0.09 |

*Note.* Non-males were the reference group. MA = mean-centered math anxiety. Residual SE = 0.04. Multiple R^2^ = 0.05, Adjusted R^2^ = 0.00, *F*(4, 80) = 1.05, *p* = .384.

^*^ indicates *p* < .05. ^***^ indicates *p* < .001.

**Table S13**

*Linear Mixed-Effects Model for Item-Level Confidence Judgments with MA by Gender by Ratio Interaction*

| Fixed Effects |  |  |  |  | Random Effects |  |
| --- | --- | --- | --- | --- | --- | --- |
|  | *b* (*SE*) | 95%CI | *t*-value | *df* |  | Variance |
| Constant | 0.12 (0.01) | [0.11, 0.14] | 18.98^***^ | 121.52 | Subject (Intercept) | 0.00 |
| Task | -0.03 (0.01) | [-0.05, -0.02] | -5.61^***^ | 83.00 |  |  |
| MA | 0.00 (0.01) | [-0.01, 0.01] | 0.67 | 143.74 |  |  |
| Gender | 0.00 (0.01) | [-0.02, 0.02] | 0.10 | 80.00 |  |  |
| SAT Math | 0.00 (0.00) | [-0.01, 0.01] | 0.75 | 80.00 |  |  |
| General Anxiety | 0.00 (0.00) | [-0.01, 0.01] | 0.32 | 80.00 |  |  |
| Task*MA | -0.00 (0.01) | [-0.02, 0.01] | -0.64 | 83.00 |  |  |

*Note.* Non-males and the passive ANS task were the reference groups. MA = mean-centered math anxiety.

^***^ indicates *p* < .001.

We replicated the main analyses with a larger sample of *N* = 131 participants (*n* = 7 outliers identified using the same criteria as reported in the main manuscript were excluded), including those who were excluded in the main manuscript due to missing standardized math scores. Because of this missing data, standardized math was not included as a covariate in these analyses. The final sample included 63 females, 60 males, and one gender non-conforming participant. As with the sample reported in the main manuscript, non-males in this sample reported higher MA (*M* = 28.50, *SD* = 5.41) than males (*M* = 26.30, *SD* = 7.09) though the difference was marginal, *t*(110) = 2.00, *p* = .05, *d* = 0.36.

**Table S14**

*Descriptive Statistics and Correlations with Confidence Intervals for Possible ANS Problems*

| Variable | *M* | *SD* | 1 | 2 | 3 | 4 | 5 |
| --- | --- | --- | --- | --- | --- | --- | --- |
|  |  |  |  |  |  |  |  |
| 1. MA (out of 45) | 27.44 | 6.36 |  |  |  |  |  |
|  |  |  |  |  |  |  |  |
| 2. ANS Passive Proportion Correct | 0.62 | 0.07 | -.29^**^ |  |  |  |  |
|  |  |  | [-.44, -.12] |  |  |  |  |
|  |  |  |  |  |  |  |  |
| 3. ANS Active Proportion Correct | 0.68 | 0.08 | .11 | .14 |  |  |  |
|  |  |  | [-.06, .28] | [-.04, .31] |  |  |  |
|  |  |  |  |  |  |  |  |
| 4. Confidence (out of 10) | 6.55 | 1.60 | -.05 | .04 | -.20^*^ |  |  |
|  |  |  | [-.22, .13] | [-.14, .21] | [-.36, -.02] |  |  |
|  |  |  |  |  |  |  |  |
| 5. Information Seeking | 13.32 | 14.42 | .04 | .08 | .44^**^ | -.07 |  |
|  |  |  | [-.14, .21] | [-.10, .25] | [.29, .58] | [-.24, .11] |  |
|  |  |  |  |  |  |  |  |
| 6. General Anxiety (out of 20) | 14.56 | 3.51 | .40^**^ | -.16 | .13 | -.14 | .04 |
|  |  |  | [.24, .54] | [-.33, .02] | [-.05, .30] | [-.31, .04] | [-.14, .22] |
|  |  |  |  |  |  |  |  |

*Note.* *M* and *SD* are used to represent mean and standard deviation, respectively. Values in square brackets indicate the 95% confidence interval for each correlation. The confidence interval is a plausible range of population correlations that could have caused the sample correlation (Cumming, 2014). MA = math anxiety. Information seeking was the number of times participants chose to click through the cells in the 2x2 array.

^*^ indicates *p* < .05. ^**^ indicates *p* < .01.

**Table S15**

*Logistic Mixed-Effect Model for Item-Level ANS Accuracy with Task by MA Interaction*

| Fixed Effects |  |  |  |  | Random Effects |  |
| --- | --- | --- | --- | --- | --- | --- |
|  | OR | *b* (SE) | 95% CI | ꭓ^2^ |  | Variance |
| Constant |  | -5.79 (0.45) | [-6.68, -4.90] | 163.98^***^ | Ratio\|Subject | 0.03 |
| Task | 1.13 | 0.12 (0.10) | [-0.09, 0.32] | 1.29 | Item (Intercept) | 0.56 |
| MA | 0.88 | -0.13 (0.04) | [-0.20, -0.05] | 10.83^**^ |  |  |
| Ratio | 236.07 | 5.46 (0.35) | [4.78, 6.15] | 242.06^***^ |  |  |
| Confidence | 0.98 | -0.02 (0.02) | [-0.05, 0.02] | 1.05 |  |  |
| Gender | 0.94 | -0.07 (0.06) | [-0.18, 0.05] | 1.30 |  |  |
| General Anxiety | 1.00 | 0.00 (0.03) | [-0.06, 0.06] | 0.02 |  |  |
| Task*Math Anxiety | 1.20 | 0.19 (0.05) | [0.10, 0.27] | 16.67^***^ |  |  |

*Note.* Non-males and the passive ANS task were the reference groups. MA = mean-centered math anxiety. AIC = 12079, BIC = 12152, Log Likelihood = -6029.

^*^ ^**^ indicates *p* < .01. ^***^ indicates *p* < .001.

**Table S16**

*Linear Mixed-Effects Model for Item-Level Confidence Judgments*

| Fixed Effects |  |  |  |  | Random Effects |  |
| --- | --- | --- | --- | --- | --- | --- |
|  | *b* (SE) | 95%CI | t-value | df |  | Variance |
| Constant | 1.42 (1.36) | [-1.24, 4.08] | 1.05 | 145.42 | Subject (Intercept) | 2.43 |
| Ratio | 3.72 (0.36) | [3.00, 4.44] | 10.21^***^ | 48.00 | Item (Intercept) | 0.43 |
| MA | -0.06 (0.16) | [-0.26, 0.38] | 0.37 | 119.00 |  |  |
| Gender | 0.58 (0.29) | [0.01, 1.15] | 2.00^*^ | 119.00 |  |  |
| ANS Passive Performance | 0.45 (2.04) | [-3.55, 4.45] | 0.22 | 119.00 |  |  |
| General Anxiety | -0.19 (0.16) | [-0.49, 0.12] | -1.18 | 119.00 |  |  |

Note*.* Non-males were the reference group. MA = mean-centered math anxiety.

^*^ indicates *p* < .05. ^***^ indicates *p* < .001.

**Table S17**

*Logistic Mixed-Effect Model for Item-Level Accuracy on Active Task Items*

| Fixed Effects |  |  |  |  | Random Effects |  |
| --- | --- | --- | --- | --- | --- | --- |
|  | OR | *b* (SE) | 95% CI | ꭓ^2^ |  | Variance |
| Constant |  | -5.95 (0.52) | [-6.97, -4.93] | 129.94^***^ | Subject (Intercept) | 0.04 |
| Information Seeking | 1.01 | 0.01 (0.00) | [0.00, 0.02] | 5.13^*^ | Item (Intercept) | 0.52 |
| Ratio | 256.50 | 5.55 (0.43) | [4.71, 6.39] | 167.75^***^ |  |  |

*Note.* AIC = 6314, BIC = 6348, Log Likelihood = -3152.

^*^ indicates *p* < .05. ^***^ indicates *p* < .001.

**Table S18**

*Logistic Mixed-Effects Model for Item-Level ANS Accuracy with Information Seeking by MA by Gender Interaction*

| Fixed Effects |  |  |  |  | Random Effects |  |
| --- | --- | --- | --- | --- | --- | --- |
|  | OR | *b* (SE) | 95% CI | ꭓ^2^ |  | Variance |
| Constant |  | -5.85 (0.53) | [-6.87, -4.82] | 125.00^***^ | Subject (Intercept) | 0.03 |
| Information Seeking | 1.00 | 0.00 (0.00) | [-0.01, 0.01] | 0.08 | Item (Intercept) | 0.52 |
| MA | 0.97 | -0.03 (0.06) | [-0.15, 0.09] | 0.20 |  |  |
| Gender | 0.85 | -0.16 (0.07) | [-0.31, -0.02] | 4.73^*^ |  |  |
| Ratio | 258.30 | 5.55 (0.43) | [4.72, 6.39] | 168.46^***^ |  |  |
| Info. Seeking*MA | 1.00 | -0.00 (0.01) | [-0.01, 0.01] | 0.04 |  |  |
| Info. Seeking*Gender | 1.02 | 0.02 (0.01) | [0.00, 0.03] | 5.36^**^ |  |  |
| MA*Gender | 1.11 | 0.11 (0.08) | [-0.05, 0.26] | 1.88 |  |  |
| Info. Seeking*MA*  Gender | 1.01 | 0.01 (0.01) | [-0.00, 0.03] | 3.44 |  |  |

*Note.* Non-males were the reference group. MA = mean-centered math anxiety. AIC = 6307, BIC = 6381, Log Likelihood = -3142.

^*^ indicates *p* < .05. ^**^ indicates *p* < .01. ^***^ *p* < .001.

**Table S19**

*Linear Mixed-Effects Model for Item-Level Information Seeking*

| Fixed Effects |  |  |  |  | Random Effects |  |
| --- | --- | --- | --- | --- | --- | --- |
|  | Estimate (*SE*) | 95%CI | *t*-value | *df* |  | Variance |
| Constant | 11.83 (12.51) | [-12.69, 36.35] | 0.95 | 115.08 | Subject (Intercept) | 199.78 |
| Ratio | -5.14 (0.71) | [-6.53, -3.75] | -7.25^***^ | 106.24 | Item (Intercept) | 2.47 |
| MA | 0.63 (1.45) | [-2.21, 3.47] | 0.43 | 114.11 |  |  |
| Gender | -1.25 (2.65) | [-6.53, 3.75] | -0.47 | 114.11 |  |  |
| Confidence | -0.51 (0.82) | [-2.12, 1.10] | -0.62 | 113.91 |  |  |
| ANS Passive Performance | 18.68 (18.31) | [-17.22, 54.57] | 1.02 | 113.97 |  |  |
| General Anxiety | 0.33 (1.42) | [-2.44, 3.10] | 0.23 | 114.05 |  |  |

*Note.* Non-males were the reference group. MA = mean-centered math anxiety.

^***^ indicates *p* < .001.
